# Common Epidemiology of *Rickettsia felis* Infection and Malaria, Africa

**DOI:** 10.3201/eid1911.130361

**Published:** 2013-11

**Authors:** Oleg Mediannikov, Cristina Socolovschi, Sophie Edouard, Florence Fenollar, Nadjet Mouffok, Hubert Bassene, Georges Diatta, Adama Tall, Hamidou Niangaly, Ogobara Doumbo, Jean Bernard Lekana-Douki, Abir Znazen, M’hammed Sarih, Pavel Ratmanov, Herve Richet, Mamadou O. Ndiath, Cheikh Sokhna, Philippe Parola, Didier Raoult

**Affiliations:** 1 These authors contributed equally to this article.; Aix Marseille Université, Marseille, Faculté de Médecine, Marseille, France (O. Mediannikov, C. Socolovschi, S. Edouard, F. Fenollar, Patmanov P., H. Richet, P. Parola, D. Raoult);; Institut de Recherche pour le Développement, Dakar, Senegal (O. Mediannikov, F. Fenollar, H. Bassene, G. Diatta, M.O. Ndiath, C. Sokhna, D. Raoult);; Centre Hospitalo-Universitaire d'Oran, Oran, Algeria (N. Mouffok); Institut Pasteur de Dakar, Dakar (A. Tall);; University of Sciences, Techniques and Technology, Bamako, Mali (H. Niangaly, O. Doumbo);; Unité de Parasitologie Médicale Centre International de Recherche Médicale de Franceville, Franceville, Gabon (J.B. Lekana-Douki);; Université des Sciences de la santé de Libreville, Libreville, Gabon (J.B. Lekana-Douki);; Habib Bourguiba University Hospital, Sfax, Tunisia (A. Znazen); Institut Pasteur du Maroc, Casablanca, Morocco (M. Sarih);; Far Eastern State Medical University, Khabarovsk, Russia (P. Ratmanov)

**Keywords:** Rickettsia felis, rickettsial, Plasmodium species, malaria, Africa, tropic, co-infection, arthropod, mosquito, febrile, fever, vector, reservoir, bacteria, parasites

## Abstract

This study aimed to compare the epidemiology of *Rickettsia felis* infection and malaria in France, North Africa, and sub-Saharan Africa and to identify a common vector. Blood specimens from 3,122 febrile patients and from 500 nonfebrile persons were analyzed for *R. felis* and *Plasmodium* spp. We observed a significant linear trend (p<0.0001) of increasing risk for *R. felis* infection. The risks were lowest in France, Tunisia, and Algeria (1%), and highest in rural Senegal (15%). Co-infections with *R. felis* and *Plasmodium* spp. and occurrences of *R. felis* relapses or reinfections were identified. This study demonstrates a correlation between malaria and *R. felis* infection regarding geographic distribution, seasonality, asymptomatic infections, and a potential vector. *R. felis* infection should be suspected in these geographical areas where malaria is endemic. Doxycycline chemoprophylaxis against malaria in travelers to sub-Saharan Africa also protects against rickettsioses; thus, empirical treatment strategies for febrile illness for travelers and residents in sub-Saharan Africa may require reevaluation.

Investigations examining the etiologic spectrum of fever of unknown origin in Africa rapidly progressed during 2008–2011 (*1**–**3*), providing increased knowledge about bacterial infections. Bacterial agents that have been most frequently identified in North and sub-Saharan Africa by culture are non-typhoidal *Salmonella*, *Streptococcus pneumoniae*, *Staphylococcus aureus*, *Escherichia coli,* and *Mycobacterium tuberculosis* (*2*). Several studies have assessed the effect of fastidious bacterial infections in systemic febrile illness, including *Rickettsia felis* (*4**–**6*), *Coxiella burnetii* (*7*), *Tropheryma whipplei* (*3*), and *Borrelia* spp. (*1**,**8*). Tourism, immigration, international business travel, international aid work, and the deployment of troops overseas were documented as contributors to a tremendous increase in international travel during 1996–2004 (*9*). International tourist arrivals reached 940 million worldwide during 2010, an increase of 6.6% over 2009, and the current total number of international migrants has increased to an estimated 214 million persons in 2012 (*10*). Consequently, physicians in the Western hemisphere increasingly encounter febrile patients returning from international travel who were exposed to tropical infections that the physicians are unfamiliar with (*9**,**10*). Among international travelers, malaria, dengue, and rickettsiosis are among the most identified etiologies of febrile illness, and exposure to mosquitoes is reported as the most common source of fever (*11*).

*Rickettsia felis,* an obligate intracellular Gram-negative bacterium belonging to the spotted fever group of *Rickettsi*a, has been shown to be a common agent of bloodstream infections in among humans Senegal and Kenya, identified in 7% of the population evaluated (*4**–**6*). However, the epidemiology (including vectors and reservoirs) and clinical picture of this emerging infection in the rest of Africa is largely unknown (*12**,**13*). During 2011, a possibly primary infection with *R. felis,* named “yaaf,” was hypothesized in the case of an 8-month-old girl in Senegal with polymorphous skin lesions (*12*). 

The considerable frequency of *R. felis* infections observed in febrile patients in malaria-endemic regions and the many relapses previously reported (*4**,**5*) led us to investigate the possible correlation of *R. felis* and that of the parasite, *Plasmodium falciparum*, a known vector of malaria. The reservoirs for malaria and many rickettsial species are mammals, including humans; humans have long been known to be a reservoir for malaria, and were documented as the reservoir for *R.* prowazekii, the agent of epidemic typhus (*14*). Vectors for both organisms are arthropods: for rickettsial diseases vectors are typically ticks, lice or mites, and infected humans are susceptible to relapse (such as epidemic and scrub typhus) (*14*).

The vectors for malaria are mosquitoes of the genus *Anopheles* that breed in warm and humid areas (*15*). Malaria is particularly common among young patients, because progressive immunity develops following multiple infections as the child grows older. Great apes in Cameroon were recently identified as targets or possibly the origin of malaria (*16*). *R. felis* has recently been detected in *Anopheles gambiae* mosquitoes in molecular form S, in *Aedes albopictus* mosquitoes, and in gorilla fecal samples (*17**–**19*). These elements suggest comparable features within the epidemiologic cycles of malaria and *R. felis* infection. In addition, co-infections by *R. felis* and *P. falciparum* have been reported in Kenya (*5*). To prove the hypothesis of the similar epidemiology of malaria and *R. felis* infection, target populations, clinical phenomena (relapses and bacteremia in apparently asymptomatic patients), and geographic and seasonal distribution should be compared. The objective of this work is to clarify the epidemiology of *R. felis* infection and to compare it with malarial epidemiology.

## Materials and Methods

### Study Areas and Participants

#### Febrile Patients

**Figure 1:**
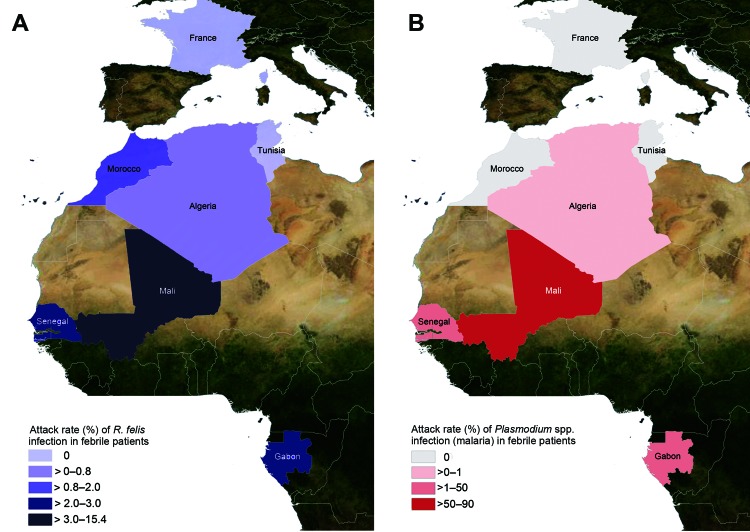
Prevalence of *Rickettsia felis* infection (A) and *Plasmodium* spp. infection (malaria) (B) in febrile patients in Gabon, Senegal, Mali, Algeria, Morocco, Tunisia, and France, June 2010–April 2012.

During June 2010‒March 2012, a cohort of 2,075 patients (67% <15 years of age; sex ratio, 1:1) from 14 health centers distributed throughout rural Senegal (Senegal study sites S_1_-S_6_) were enrolled in this study. The study sites spanned various ecosystems, from dry regions in the north (Dielmo, Senegal study region 1, S_1,_ Ndiop-S_2_, Keur Momar Sarr–S_3_, and Niakhar-S_4_) to humid regions in the south (Basse-Casamance-S_5_ and Kedougou-S_6_) that had a rainy season during June through October (Technical Appendix Table). In addition, patients from various medical facilities were included: 100 from rural Mali dispensaries: Diankabou-Mali study site M_1_ and Kole-Mali study site M_2_; 50 from Franceville, in urban Gabon (pediatric consultation); 183 from Sfax, Tunisia (infectious diseases and pediatric departments); 266 from Oran, Algeria (department of infectious diseases); 48 from the Kenitra region, rural Morocco (dispensaries); and 400 from Marseille, France (hospital emergency units) (Figure 1). Questionnaires and informed consent forms were completed upon enrollment in the study. For each febrile patient (axillary temperature >37.5°C), an interview was conducted, a blood sample (200 µl blood containing EDTA) was collected, and a medical examination was performed. The national ethics committees of Senegal, Gabon, and France approved this project (No. 0–00.87MSP/DS/CNERS and No. 001380MSP/DS/CNERS). 

#### Control Group

Samples were obtained from 400 afebrile persons (62% >15 years of age) from S_1–2_ who participated in a longitudinal study of malaria (*20*) and 100 persons from France who were under the medical care of 1 of the authors (D.R.) for conditions other than malaria.

### Arthropod Collection in Senegal

Arthropod specimens collected in Senegal consisted of 949 adult mosquitoes from 3 locations (Table 1, 154 mosquito larvae from Mariste, Dakar, 370 ticks from 2 locations, 160 adult bed bugs from 6 locations, and 384 midges from 2 locations. The *Anopheles arabiensis* mosquito larvae were collected from breeding sites in Mariste, Dakar. The pooled larvae were maintained under laboratory conditions until they grew to the adult stage. In sites S_1–2_, 144 adult ticks (2 *Rhipicephalus* spp., 4 *Argas persicus*, and 138 *Ornithodoros sonrai*) from 55 burrows inside of 16 human dwellings were collected. A total of 226 *Ornithodoros capensis* ticks were manually collected from the nests of great cormorants (*Phalacrocorax carbo*) in Sarpan Island (îles de la Madeleine) near Dakar. Bed bugs were manually captured from the beds of ill persons. The collection of *Culicoides* spp. was performed in S_1–2_ by using overnight posed CDC light traps with 0.7-mm mesh size. The arthropods were identified at the species level by using morphological characteristics according to identification keys.

**Table 1:** Detection of *Rickettsia felis* DNA in mosquitoes, Senegal. March 2010–September 2012

Geographic location in Senegal	Period of collection	Collection method	Mosquito species morphological identification	DNA samples	*Rickettsia felis* detection (%)
Ferlo, 15°52′N, 15°15W	Mar 2010	CDC type light trap collections*	*Aedes luteocephalus*	203	1 (<1)
			*Culex quinquefasciatus*	186	0/186
Dakar, 14°41′N, 17°26W	Dec 2011	Immature stages-lab conditions	*Anopheles arabiensis*	154 †	2 (<1)‡
Dielmo, 13°43′N, 16°24′W	Jul 2012	Human landing catches	*An. arabiensis*	8	0
			*An. welcomei*	6	0
			*Mansonia uniformis*	6	0
			*C. quinquefasciatus*	4	0
	Sep 2012	Pyrethrum spray catches	*An. ziemanni*	7	1 (14)
			*An. pharoensis*	10	1 (10)
			*M. uniformis*	8	2 (25)
			*An. welcomei*	8	0
			*An. funestus*	7	2 (29)
Elinkine, 12°30′N 16°39′W	Sep 2012	CDC-type light trap collections	*An. gambiae*	50	0
			*Culex* sp.	10	0
		Pyrethrum spray catches	*Culex* sp.	10	0
		Human landing catches	*An. gambiae*	290	0
			*An. squamosus*	27	0
			*An. ziemanni*	31	0
			*Culex* sp.	35	0
			*Aedes* sp.	23	0
			*Mansonia* sp.	20	0
Total				1,103	9 (<1)

*Manufactured by John W. Hock Company, Gainesville, FL, USA. † including 20 male mosquitoes.  ‡ *R. felis* DNA was detected in 1 male mosquito.

### Molecular Analysis

DNA was extracted by using the 2-stage protocol for a QIAamp kit (QIAGEN, Hilden, Germany) for the S_4–6_ groups (*3**,**4**,**7*), and a Biorobot EZ1 Workstation (QIAGEN, Courtaboeuf, France) was used to extract DNA from samples from S_1–2_, Algeria, Tunisia, Morocco, and France. In Gabon, the DNA Blood Omega Bio-tek-E.Z.N.A method (Omega Bio-tek, Norcross, GA, USA) was used according to the manufacturer’s protocol. For all locations, DNA was eluted in 100 µL of elution buffer, and 5 µL was used per reaction.

Quantitative real-time reverse transcription PCR (qRT-PCR) was performed by using a 7900HT-thermocycler (Applied Biosystems) with the QuantiTect-Probe PCR Kit (QIAGEN, Courtabeuf, France). Only samples positive for the β-actin gene product were considered reliable (*3*); thus, 51 and 9 samples from Senegal and Algeria, respectively, were excluded. All samples were screened by using a *Rickettsia* genus-specific qRT-PCR targeting the *gltA* gene and an *R. felis*-specific qRT-PCR targeting the *bioB* gene (*4*). The positive samples were tested by a second *R. felis*-specific qRT-PCR targeting the *orfB* gene (*18*). A sample was considered positive when the qRT-PCRs were positive for the 2 different specific genes. Positive samples from arthropods were further tested for plasmid pRFδ (*21*) and by a newly designed *R. felis*-specific qRT-PCR targeting the *vapB1* gene with the primers VapB1.R (5′-AGGCGAAAGCTTTGACGTG-3′) and VapB1.F (5′-TGTCTTTCATGAATTGATCAGCA-3′) and the probe VapB1.P (6-FAM-5′-AAGGCTTGGTTTCTGCGGGC-3′TAMRA).

Blood smears stained with Giemsa were examined for the samples collected in Gabon. All other samples were tested by using a *Plasmodium-*genus specific qRT-PCR targeting the *Cox*-1 gene found in all *Plasmodium* species; the primers Psp_15.F (5′-AGGAACTCGACTGGCCTACA-3′) and Psp_16.R (5′-CCAGCGACAGCGGTTATACT-3′) and the (6FAM-5′-CGAACGCTTTTAACGCCTGACATGG-3′TAMRA) probe were used. The positive samples were subsequently tested by *Plasmodium*-genus specific qRT-PCR targeting 18S rRNA with the primers Plasmo_18S_2_MBF (5′-AGGCAACAACAGGTCTGTGA-3′) and Plasmo_18S_2_MBR (5′-GCAATAATCTATCCCCATCACG-3′) and the (6FAM-5′- GAACTAGGCTGCACGCGTGCTACA-TAMRA-3′) probe.

### Statistical Analysis

Statistical analyses were performed by using the Statcalc module of Epi Info 3.5.3 (Centers for Disease Control and Prevention, Atlanta, GA, USA) to calculate the χ^2^ values for the incidence rate trends calculated for each country. PASW Statistics software 17.0 (IBM, SPSS Inc., Armonk, NY, USA) was used to perform Pearson correlation analyses. The relative risk (RR) and the 95% CI of the risk were calculated by using either the Mantel-Haenszel χ^2^ test or Fisher’s exact test. The statistical significance of the χ^2^ values was evaluated at α = 0.05. The attack rates of *R. felis* infection and malaria were calculated for each country, site, sex, and age range. In contrast, the incidence rates of *R. felis* infection and malaria for S_1–2_ were calculated monthly and yearly from June 13, 2010 through October 13, 2011. The data from a study performed in 2009 (*4*) were combined with those of this study to determine the frequency of relapses or re-infections of *R. felis* infections in S_1–2_.

## Results

### *Rickettsia felis* Detection

#### Senegal

 The attack rate of *R. felis* infections in febrile patients was 15% (312/2,024); those infections occurred primarily during the rainy season rather than the dry season (207/1,105 vs. 105/916, respectively; p<0.0001). The risk of developing *R. felis* infection was 1.6× higher during the rainy period (95% CI 1.3–2) than during the dry period. When calculated by site, substantial differences in the rates of *R. felis* infection were observed (Table 2). The highest attack rates were observed in S_5–6_, reaching 40% (92/231) from August‒October 2011. The lowest attack rate was observed in S_1–2_ (7%–8%) and was significantly lower than that observed at the 4 other sites S_3–6_ (p≤0.001) (Table 2).

**Table 2:** Attack rate of *Rickettsia felis* infection and malaria by country and geographic site, Africa, 2010–2012

					No. samples positive/no. tested (%)		
Participant status Country Study site (site abbreviation)	Collection period			No. samples*	*R. felis *	*Plasmodium* spp.	*R. felis/* *Plasmodium* spp. co-infection
Febrile patients								
Senegal	Jun 2010–Mar 2012		2,024		312/2,024 (15)	400/1,867† (21)	66/285 (23)*	
Dielmo (S_1_)	Jun 2010–Feb 2012		540		39/540 (7)	118/509 (23)	8/36 (22)	
Ndiop (S_2_)	Jun 2010–Feb 2012		246		20/246 (8)	33/237 (14)	3/18 (17)	
Keur-Momar Sarr (S_3_)	Mar–Nov 2011		223		36/223 (16)	44/196 (22)	9/33 (27)	
Niakhar (S_4_)	Oct 2010–Mar 2012		316		76/316 (24)	74/303 (24)	18/74 (24)	
Basse-Casamance (S_5_)	Jan 2011–Mar 2012		411		84/411 (20)	37/350 (11)	7/69 (10)	
Kedougou (S6)	2011		288		57/288 (20)	94/272 (34)	21/55 (38)	
Gabon								
Franceville	2011		50		5/50 (10)	19/50 (38)‡	2/5 (40)**	
Mali	2011		100		3/100 (3)	90% (90/100)	3/3 (100)	
Diakambou (M1)	Oct		50		1/50 (2)	82% (41/50)	1/1 (100)	
Kole (M2)	Nov		50		2/50 (4)	98% (49/50)	2/2 (100)	
Algeria								
Oran	Jul–Sep 2012		257		2/257 (1)	1/257 (0,4%)	0/1	
Morocco								
Casablanca	May–Jun 2006		48		1/48 (2)	0/38†	0	
Tunisia								
Sfax	2012		183		0/183	0/183	0	
France								
Marseille	2012		400		0/400	0/400	0	
Afebrile persons Senegal (S_1_-S_2_)	Dec 20s11–Apr 2012	391			17/391 (4)	5/391 (1)	0/5	
France								
Marseille	2011–2012	100			0/100	0/100	0	

*Reliable samples. †There were insufficient DNA samples for the analysis of *Plasmodium* spp., as decided a posteriori. ‡Positive by blood smear.

**Figure 2:**
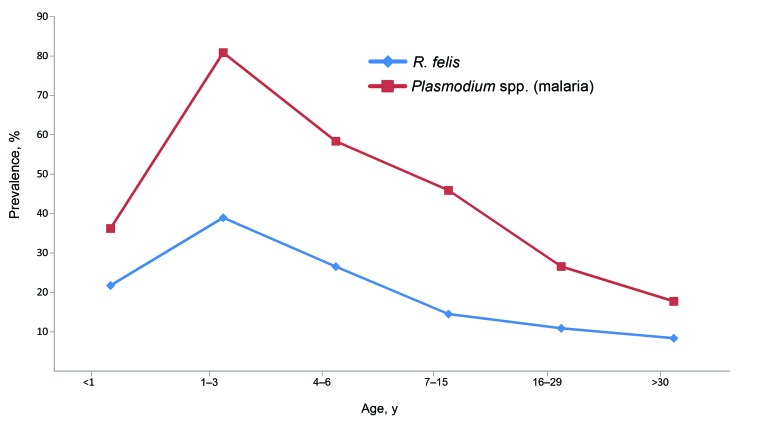
Incidence of *Rickettsia felis* and *Plasmodium* spp. infection (malaria) in patients, by age, in Dielmo and Ndiop, Senegal.

Incidence rates were obtained from 2 health centers (Figure 2). In 2011, the incidence rate of *R. felis* in S_1_ was 6.7 (4.8‒9.0) per 100 person-years or 0.55 (0.39‒0.76) per 100 person-months; the incidence rate in S_2_ was 3.1 (1.8‒4.9) per 100 person-years or 0.26 (0.15‒0.41) per 100 person-months during the same period. In S_1–2_, a significant difference was found between the incidence of *R. felis* for patients <15 years of age, which was 0.23 (0.16‒0.31) per 100 person-months, and the incidence in patients >15 years of age, which was 0.10 (0.06‒0.15) per 100 person-months (relative risk [RR] 2.38, 95% CI 1.34–4.28, p = 0.003). When the incidence rates by age group were calculated according to sex, a significant difference was observed only in the male group, in which the incidence rate was significantly higher in the patients <15 years of age than in the patients >15 years of age (0.29 vs. 0.07 per 100 person-months, RR 5.97, 95% CI 2.28–17.15, p = 0.001).

Table 3 shows the age distribution of *R. felis* infection. The occurrence of *R. felis* infection was significantly lower in patients 1–3 years of age (10%) than in patients >4 years of age (p = 0.03 for patients 4‒6 years of age (15%); p = 0.003 for patients 7‒15 years of age (16%); p = 0.004 for patients 16 to 29 years of age (16%); p = 0.002 for those >30 years of age (17%). The sex ratio for *R. felis* was 145M/162F (1:1.1). No deaths associated with *R. felis* infection were registered.

**Table 3:** Age distribution of infections with *Rickettsia felis* and *Plasmodium* spp. among patients in Senegal, by age group, 2010-2012

Species	No. positive samples/no. tested samples (%)							
	<1 y	1–3 y	4–6 y	7–15 y	16–29 y	>30 y	ND	Total
*Plasmodium* spp.	20/169 (12)	60/327 (16)	57/251 (23)	134/401 (33)	56/271 (21)	58/310 (19)	15/78 (19)	400/1,867 (21)
*R. felis*	27/184 (15)	40/422 (10)	40/270 (15)	69/425 (16)	49/298 (16)	57/336 (17)	30/89 (34)	312/2,024 (15)

*NO, age data available.

Combining these data with our preliminary report of 8 infected patients during 2008–2009 in S_1–2_ (*4*), we identified 61 patients with *R. felis* infections among a total of 456 villagers tested in S_1–2_. A second *R. felis* infection was diagnosed in 5 patients after 44 to 911 days, and 1 patient was positive for *R. felis* infection a second and third time at days 378 and 441, respectively. The 6 patients (4 male, 2 female) who had relapses or re-infections were from S_1_, and 5 were <6 years of age.

#### Other Countries

 Samples from 3 patients (3%, 3/100) in rural Mali (M_1_, 1/50; M_2_, 2/50), 5 patients (10%, 5/50) in urban Gabon, 1 patient (2%, 1/48) in rural Morocco, and 2 patients (1%, 2/257) in Algeria were positive for *R. felis* (Table 2). Conversely, *R. felis* DNA was not detected among the samples from febrile persons in France and Tunisia.

When the *R. felis* infection rates of the different countries were compared, *R. felis* was detected more often in countries with high malaria rates compared with countries with low malaria rates (Senegal, Gabon, and Mali vs. Algeria, Tunisia, Morocco, and France; p<0.001) (Figure 1). The trend analysis showed a significant linear trend of increasing risk for *R. felis* infection; a lower risk was shown in northern countries (France, Tunisia, Morocco, and Algeria) and a higher risk in southern countries (Mali, Gabon, and Senegal) (p < 0.0001). The probability of *R. felis* infection was 1.00 for Algeria (baseline), 2.8 for Morocco, 4 for Mali, 14.5 for Gabon, and 24 for Senegal.

### Malaria

#### Senegal

The attack rate of *Plasmodium* spp. in febrile persons from Senegal was 21% (400/1868, 206 females); those infections occurred significantly more often during the rainy season compared with the dry season (256/1042 vs. 144/822, respectively; p = 0.0002). The risk for malaria was 1.4× higher during the rainy period than during the dry period (95% CI 1.2–1.7, p<0.0001). The highest rate was in southeastern S_6_, whereas the lowest rate, 11% (37/350), was in southwestern S_5_ (Table 2). During the same time period, the incidence rate of malaria was 17.6 per 100 person-years or 1.46 per 100 person-months for S_1_ and 5.1 per 100 person-years or 0.42 per 100 person-months for S_2_. The highest incidence of malaria was among patients <15 years of age in S_1–2_ (0.55 (0.44–0.67) versus 0.22 (0.16–0.30) per 100 person-months, RR 2.51, 95% CI 1.73–3.65, p<0.0001).

When the incidence rate by age group was calculated according to sex, the highest incidence was found among girls <15 years of age: 0.47 (0.37–0.65) versus 0.25 (0.16–0.37) per 100 person-months, RR 1.92, 95% CI 1.15–3.18, p = 0.01; and boys (0.62 (0.47–0.81) versus 0.18 (0.10–0.30) per 100 person-months, RR 3.38, 95% CI 1.90–5.99, p<0.0001) groups. Table 3 shows the age distribution of malaria. Patients 7‒15 years of age (33%) were infected with *Plasmodium* spp. significantly more often than those in other age groups (15% for patients 1‒3 years of age, p<0.0001 to 23% for patients 4‒6 years of age, p = 0.004). Co-infection of *Plasmodium* spp. and *R. felis* was found in 66 case-patients (23%, 66/285), mostly in women (61%) and in children 7‒15 years of age (43%).

#### Other Countries

*Plasmodium* DNA was detected in 90% of the blood samples collected in Mali; 3 patients with malaria from Mali were co-infected with *R. felis* (Table 2). In Gabon, samples from 38% (19/50) of the patients tested positive for malaria by using blood smears; 2 of those patients were co-infected with *R. felis*. We most likely misdiagnosed malaria among the patients in Gabon, as based on the lower sensitivity and high specificity of microscopy versus PCR as the standard (*22*). *Plasmodium* DNA was not detected in the samples from Tunisia, Morocco, or France. However, 1 *Plasmodium* spp.-positive sample was collected in Algeria from a 21-year-old woman who was hospitalized for high fever, chills, and sweats after having spent >2 months visiting her family in Niger without malaria chemoprophylaxis.

### Correlation of *R. felis* with Malaria

Using the Pearson correlation test, we found a significant correlation between the number of patients infected with *R. felis* and those infected with *Plasmodium* spp. (p<0.002): a higher number of *R. felis* infections correlated with a higher number of malaria cases. A significant correlation was also found for seasonality for infection by both pathogens: most cases occurred during the rainy period (p<0.0001). In addition, children <3 years of age were infected with both organisms less often than persons >4 years of age, and the Pearson test showed a significant correlation between *R. felis* and malaria (p = 0.001) for this age group.

#### Control Group

*R. felis* DNA was detected in 4% of the afebrile persons (17/391) from Senegal, 12 of whom were children (<15 years of age); malaria was detected in 5 afebrile persons, 3 of whom were children. Both pathogens were detected significantly less often in afebrile patients than in febrile patients (p<0.001). DNA from *R. felis* and *Plasmodium* spp. were not detected among persons in the control group in France.

### Arthropod Study

Samples from 9 mosquitoes (≈1%, 9/1,103) and 1 bed bug (≈1%, 1/160) tested positive in 2 *R. felis*-specific qRT-PCRs (Tables 1,4). The pRFδ plasmid was detected in 8 mosquito samples (*21*). In Dakar, 1% (2/154) of the *An. arabiensis* mosquitoes collected were positive for *R. felis,* including 1 male, suggesting transovarian transmission. One *Aedes luteocephalus* from Ferlo 0.5% (1/203) was positive for *R. felis*. In S_1_, 15% (6/40) mosquitoes collected in September 2012 were positive for *R. felis*, including 1 *An. ziemanni,* 1 *An. pharoensis*, 2 *Mansonia uniformis,* and 2 *An. funestus*. None of the 24 mosquito samples collected from this region in July tested positive. In addition, 1 *Cimex hemipterus* bed bug (3%) (1/39), collected from a household in S_1_ in February 2012, tested positive. No *R. felis* DNA was detected in soft or hard ticks or in *Culicoides* species.

**Table 4:** Detection of *Rickettsia* species in arthropods collected in Senegal, 2008–2012

Group	Species	No. samples tested	Type of rickettsia (% positive samples)	Reference
Fleas	*Ctenocephalides felis*	48	None	(*13*)
	*Echidnophaga gallinacea*	150	None	
	*Synosternus pallidus*	41	*Rickettsia* sp., group *R. felis* (93)	
Tsetse flies	*Glossina morsitans submorsitans*	78	*Rickettsia* sp., group *R. felis* (100)	(*30*)
Hard ticks	*Amblyomma variegatum*	492	*Rickettsia africae* (87)	(*29*)
	*Rhipicephalus decoloratus*	40	Rickettsiae spotted fever group (0–51)	(*27*)
	*R. annulatus*	5		
	*Hyalomma marginatum rufipes*	173		
	*H. truncatum*	141		
	*R. evertsi evertsi*	2358		
	*R. guilhoni*	50		
	*Rhipicephalus* sp.	2	None	This study
Soft ticks	*Ornithodoros sonrai*	138	None	This study
	*O. capensis*	40	*Rickettsia* sp., group *R. felis* (20)	This study
	*Argas persicus*	4	None	This study
Midges	*Culicoides* spp.	384	None	This study
Bed bugs	*Cimex hemipterus*	160	1/160, (0.6)	This study

## Discussion

This study shows that *Rickettsia felis* is an emerging pathogen commonly detected in sub-Saharan rural Africa. We are confident that our molecular results are reliable and that the negative results in samples from France illustrate a correlation between *R. felis* infection and malaria with regard to the geographic distribution and seasonality. A trend of higher risk for *R. felis* infection in southern countries than in northern countries was revealed; the highest risk for *R. felis* infection was in rural Senegal (24 times than in Algeria). In Senegal, DNA from *Plasmodium* spp. and *R. felis* were detected at high levels, mostly during the rainy season and among children <15 years of age (Figure 2), but no coincidental relationship was found. The incidence of co-infection of *R. felis* and malaria was lower in Senegal (23%) than in Kenya (79%) (*5*), but higher than the rate of simultaneous bacterial bloodstream infections and malaria parasitemia, which ranged from 6% in rural Mozambique (*23*) to 11% in Nairobi (*24*). Mixed infections for rickettsioses, including co-infections with malaria or with other bacteria (*Leptospira* spp., *Coxiella burnetii*, and *Burkholderia pseudomallei*) have been described (*25*).

*R. felis* was detected in afebrile persons, most of whom were children <15 years of age, confirming the previously reported results in Kenya (*5*). Although rickettsioses have not previously been reported in afebrile persons, low-grade *Plasmodium* parasitemia has been reported among persons without a fever (*26*). This result should be confirmed by culture, but *R. felis* has never been isolated, even from acutely ill patients. Nonetheless, the absence of positive tests in the control group located in France confirmed the specificity of our tests. The S_1–2_ population was screened serologically for *R. felis*, and low titers were identified in 1 of 479 serum samples tested (*27*), which is substantially lower than the seroprevalence of other spotted fever group rickettsiae. The mechanism of absence of a serologic response and the occurrence of multiple re-infections or relapses of *R. felis* should be investigated further.

In this work, we demonstrated a greater frequency of *R. felis* during the rainy season among children in the subtropical zones, a period coinciding with circulation of *P. falciparum*. There are other seasonal diseases, including influenza, which are most common during the rainy season in subtropical Africa, particularly in Senegal (*28*). Influenza is a disease found throughout the year, with seasonal peaks, in Africa; none of the tested patients had influenza symptoms. Furthermore, leptospirosis, for which rickettsial disease could be mistaken, has not been documented in Senegal. Last, the most common seasonal disease in the most northern part of the intertropical area is malaria; a disease, however, which is common in all seasons in equatorial wetlands. These data, for which confirmation is needed, show a seasonal correlation between *R. felis* and malaria; the correlation is related to the presence and activity of *Anopheles* mosquitoes. Although the cat flea, *Ctenocephalides felis*, is currently the only known vector of *R. felis*, a variety of other arthropods have been suspected, including different flea species, ticks, mites, and lice (*13*). In Senegal, the source of *R. felis* is yet to be determined. We did not detect *R. felis* in fleas that were screened during 1 year in S_1_ and S_2_ (*13*). In other studies, *R. felis* was not detected in soft or hard ticks (*27**,**29*), tsetse flies (*30*), or midges. These findings support the hypothesis of the role of *Anopheles* in the transmission of *R. felis*; this hypothesis should be confirmed or refuted by future studies.

The clinical findings for *R. felis* infection are often unclear and are typically misdiagnosed as other febrile illnesses (*12**,**31*). Recently, the primary infection was described in a patient with polymorphous skin lesions, including papules, vesicles, erosions, and ulcers (*12*), similar to patients from Mexico (*32*). In the current study, a high incidence of *R. felis* infection was identified in children <15 years of age, as described (*4*). Fortunately, such patients improve rapidly with doxycycline treatment (*12*). For travelers to sub-Saharan Africa, the medications recommended for the chemoprophylaxis of malaria include doxycycline, which has the added advantage of being effective against rickettsioses (*33*).

This study showed the wide distribution and high incidence of *R. felis* infection; therefore, rickettsiosis should be considered one of the major causes of febrile diseases in sub-Saharan Africa. The demonstrated geographic distribution, seasonality, target population, incidence of relapses or re-infections, and asymptomatic infections of *R. felis* infection are similar to malaria. Further studies are needed to investigate the hypotheses that humans, as for epidemic typhus, another vector-borne relapsing rickettsiosis, or apes could be reservoirs and mosquitoes could be a vector for *R. felis* infection.

Technical Appendix Demography, geography, and climate of the rural regions studied for comparison of detection of *Rickettsia felis* and *Plasmodium* spp.
