# Supplementary material for: Common Epidemiology of Rickettsia felis Infection and Malaria, Africa
Source: Emerg Infect Dis. 2013 Nov;19(11):1775–83. doi: 10.3201/eid1911.130361 (PMC3837673; doi:10.3201/eid1911.130361)
Supplement: Technical Appendix — Demography, geography, and climate of the rural regions studied for comparison of detection of Rickettsia felis and Plasmodium spp. [file 13-0361-Techapp-s1.pdf]

Article DOI: http://dx.doi.org/10.3201/eid1911.130361

# Common Epidemiology of *Rickettsia felis* Infection and Malaria, Africa

## Technical Appendix

Technical Appendix Table. Rural health centers and laboratories that participated in study of epidemiology of *Rickettsia felis* Infection and malaria, featuring the demography, geography, and climate of the study regions*

| Country and study site (site abbreviation) | Region/department/district | Population size | Climate,vegetation | Annual precipitation, mm | Village | Coordinates | Other activities |
|---|---|---|---|---|---|---|---|
| Senegal<br>Dielmo-Ndiop ($S_1$-$S_2$) | Toubacouta/Foundiougne/Fatick | 120,554 | Sudanian†, wooded savannah | 939 | Dielmo | 13°43′N, 16°24′W | Point-of-care laboratory |
| | | | | | Ndiop | 13°41′N, 16°23′W | None |
| Keur Momar Sarr ($S_3$) | Keur Momar Sarr/Louga/Louga | 70,743 | Sahelian‡,steppe-type | 400 | Keur Momar Sarr | 15°55′N, 15°58′W | DNA extraction |
| | | | | | Loboudou | 15°57′N, 15°55′W | None |
| | | | | | Ganket Balla | 15°58′N, 15°55′W | None |
| Niakhar ($S_4$) | Niakhar/Fatick/Fatick | 69,446 | Sahelo-Sudanian§, wooded steppe | 757 | Toucar | 14°32′N, 16°28′W | None |
| | | | | | Diohine | 14°30′N, 16°30′W | None |
| | | | | | Ngayokheme | 14°32′N, 16°26′W | None |
| | | | | | Niakhar | 14°28′N, 16°23′W | DNA extraction |
| Basse Casamance ($S_5$) | Loudia-Oulof/Oussouye/Ziguinchor | 57,505 | Sub-Guinean¶, primary and secondary gallery forests | 1,432 | Mlomp | 12°33′N, 16°34′W | DNA extraction |
| | | | | | Kagnout | 12°33′N, 16°37′W | None |
| | | | | | Elinkine | 12°30′N, 16°39′W | None |
| Kedougou ($S_6$) | Bandafassi/Kedougou/Kedougou | 20,021 | Sudano-Guinean#, woodland, wooded savannah | 1,189 | Bandafassi | 12°32′N, 12°18′W | DNA extraction |
| | | | | | Ibel | 12°30′N, 12°22′W | None |
| | | | | | Tiabeji | 12°38′N, 12°25′W | None |

| Country and study site (site abbreviation) | Region/department/district | Population size | Climate,vegetation | Annual precipitation, mm | Village | Coordinates | Other activities |
|---|---|---|---|---|---|---|---|
| Mali<br>Diankambou ($M_1$) | Mopti | 11,333 | Sahelian, savannah | 580 | – | 14°35′N; 3°05′W | DNA extraction |
| Kole–$M_2$ | Sikasso | 3,508 | Sudano-Guinean, savannahs, forest | 700–1,500 | – | 10°5′N; 7°20′W | DNA extraction |
| Gabon<br>Franceville (G) | Mpassa/Haut-Ogooué | 56,000 | Equatorial**, savannah and tropical forest | 1,862 | – | 1°37′S; 13°34′E | DNA extraction |
| Algeria<br>Oran | Oran Province | 18,682,000 | Semi-arid climate, halophytic vegetation | 326 | – | 35°41′N 0°37′W | None |
| Morocco<br>Kenitra | Gharb-Chrarda-Béni Hssen | 1,859,540 | Mild Mediterranean climate, woody flora | 750 | Sidi Mohamed Lahmar<br>Sidi Taybi | 34°15′N 6°35′W | None |
| Tunisia<br>Sfax | Sfax Governorate | 2,256,320 | Mediterranean climate, wild grasses | 465 | – | 36°48′N 10°11′E | None |
| France<br>Marseille | Provence-Alpes-Côte d'Azur | 1,582,000 | Mediterranean climate, Mediterranean maquis shrubland†† | 515 | – | 43°17′N 5°22′E | DNA extraction |

*–, no participants at village level; NA, not applicable.
†Sudanian, tropical semi-arid climate with a mean annual precipitation between around 600 and 800 mm.
‡Sahelian, tropical semi-arid climate with a dry season mainly during the coldest 6 months of the year and a mean annual precipitation ≈200–400 mm.
§Sahelo-Sudanian tropical semi-arid climate with a mean annual precipitation ≈400–600 mm.
¶Sub-Guinean, tropical monsoon maritime climate characterized by the changing of dry and wet seasons; mean annual precipitation rate ≈1200–1500 mm.
#Sudano-Guinean, transitory climate between Sudanian and sub-Guinean climates; annual precipitation ≈ 800–1200 mm.
**Equatorial, a climate with no dry season; mean precipitation values of all months are ≥60 mm.
††Maquis, a shrubland biome in the Mediterranean region, typically consisting of densely growing evergreen shrubs such as holm oak, Kermes Oak, tree heath, strawberry tree, sage, juniper, buckthorn, spurge olive. and myrtle.
